# Comparative and Evolutionary Analyses on the Complete Plastomes of Five *Kalanchoe* Horticultural Plants

**DOI:** 10.3389/fpls.2021.705874

**Published:** 2021-08-27

**Authors:** Xiangyu Tian, Jia Guo, Xiaojiao Zhou, Ke Ma, Yonggui Ma, Tuansheng Shi, Yuhua Shi

**Affiliations:** ^1^School of Life Sciences, Zhengzhou University, Zhengzhou, China; ^2^Key Laboratory of Medicinal Animal and Plant Resources of Qinghai-Tibetan Plateau, Qinghai Normal University, Xining, China

**Keywords:** complete plastid genomes, *Bryophyllum*, *Kalanchoe*, phylogenetic relationship, positive selection

## Abstract

Many species of the genus *Kalanchoe* are important horticultural plants. They have evolved the Crassulacean acid metabolism (CAM) photosynthetic pathway to allow them to be better adapted to dry environments. Despite their importance, it is still debating whether *Kalanchoe* is monophyletic, and understanding the past diversification of this genus requires a tremendous amount of effort and work being devoted to the studies of morphological and molecular characters of this genus. However, molecular information, plastic sequence data, in particular, reported on *Kalanchoe* species is scarce, and this has posed a great challenge in trying to interpret the evolutionary history of this genus. In this study, plastomes of the five *Kalanchoe* species, including *Kalanchoe daigremontiana, Kalanchoe delagoensis, Kalanchoe fedtschenkoi, Kalanchoe longiflora*, and *Kalanchoe pinnata*, were sequenced and analyzed. The results indicate that the five plastomes are comparable in size, guanine-cytosine (GC) contents and the number of genes, which also demonstrate an insignificant difference in comparison with other species from the family Crassulaceae. About 224 simple sequence repeats (SSRs) and 144 long repeats were identified in the five plastomes, and most of these are distributed in the inverted repeat regions. In addition, highly divergent regions containing either single nucleotide polymorphism (SNP) or insertion or deletion (InDel) mutations are discovered, which could be potentially used for establishing phylogenetic relationships among members of the *Kalanchoe* genus in future studies. Furthermore, phylogenetic analyses suggest that *Bryophyllum* should be placed into one single genus as *Kalanchoe*. Further genomic analyses also reveal that several genes are undergone positive selection. Among them, 11 genes are involved in important cellular processes, such as cell survival, electron transfer, and may have played indispensable roles in the adaptive evolution of *Kalanchoe* to dry environments.

## Introduction

The genus *Kalanchoe* Adans. in the family Crassulaceae comprises 144 species (Kubitzki, 2007) and are mainly distributed in the tropical regions of Africa and Asia. Members of this genus typically have succulent leaves, pendulous or erect flowers, and eight stamens inserted in the middle or at the base of the tubular corolla (Baldwin, 1938). Many *Kalanchoe* species are important horticultural plants for they can be readily propagated from stem cuttings or clonally produced as small plantlets along the leaf margins. When the genus *Kalanchoe* was first published by Michel Adanson (1727–1806), its taxonomical status with *Bryophyllum* Salisb. and *Kitchingia* Baker was not clear (Baldwin, 1938). With advances in molecular technology, the taxonomical status of *Kalanchoe* has been revisited, and progress has been made in this area in recent years (Van Ham and Hart, 1998; Gehrig et al., 2001; Gontcharova and Gontcharov, 2009; Chernetskyy, 2013; Smith and Figueiredo, 2018). However, most of the work focused on just a few molecular markers, such as *rbcL* (14 species) and *matK* (12 species). Based on a few evolutionary studies on the internal transcribed spacer (ITS) sequences in *Kalanchoe* (Gehrig et al., 2001), *Kalanchoe* has diverged into three groups (*Kalanchoe, Bryophyllum*, and *Kitchingia*). However, uncertainty remains, and more work needs to be done to fully elucidate the taxonomical properties and the phylogenetic history of *Kalanchoe*.

The three-section (sect. *Kalanchoe*, sect. *Bryophyllum*, and sect. *Kitchingia*) view of *Kalanchoe* (Boiteau and Allorge-Boiteau, 1995) is rather popular and well-accepted. Members of the species from these sections demonstrate differences in flower morphology and geographical distribution. Species of the sect. *Kalanchoe* tend to have erect flowers. Stamens are connate and located in the middle of the tubular corolla. Members of the sect. *Bryophyllum* usually bears bulbils along their leaf margins. Flowers are pendent, and sepals fuse to form an inflated tube. Stamens are found at the base of the corolla tube. Species of the sect. *Kitchingia* share the same flower morphology with the sect. *Bryophyllum* and the same stamen position with the sect. *Kalanchoe*, with only one distinction in its floral structure, the spreading of the carpels. Due to the presence of similarities and variations both within and across sections, morphology alone may not be sufficient in putting these species into the right taxonomical groups.

The family Crassulaceae contains both C_3_ and C_4_ plant species, and it is evident that these C_4_ species could have evolved from C_3_ ancestors by independently acquiring characters required by a C_4_ system (Christin and Osborne, 2014; Yang et al., 2017). Initially, the ecological distribution of a C_4_ species is largely determined by the ecological preference of its C_3_ ancestor. However, as adaptive characters accumulate, the C4 species can “escape and radiate” in a completely new habitat. The sect. *Kalanchoe* species are widely distributed in tropical Africa and Asia, representing an extremely dry habitat, whereas sect. *Bryophyllum* and sect. *Kitchingia* are endemic to Madagascar, a more humid site (Gehrig et al., 2001). The molecular phylogenetic analyses of *matK* and ITS suggest that *Kalanchoe* might originate from an allopolyploid C_3_ species in humid regions of Madagascar, and then spread into arid habitats (Baldwin, 1938; Gehrig et al., 2001, Mort et al., 2001). To adapt to a drier environment, *Kalanchoe* species have probably gained some drought-resistant traits, such as the coordination of leaves and the CAM photosynthetic pathway, which are enciphered in their genomes and/or plastomes (Yang et al., 2017). To fully understand how *Kalanchoe* has evolved to become adaptive to arid habitats, as much sequence data as possible should be collected and analyzed.

With the rapid development of next-generation sequencing technology, a large number of genomic sequence data from different taxonomic groups have been obtained and used for phylogenetic studies (Palmer and Stein, 1986; Daniell et al., 2016). Due to the lack of recombination and the ease of amplification, the highly conserved plastid genomes have been widely used to reveal the phylogenetic relationship and evolutionary dynamics among different taxonomic groups (Jansen et al., 2007; Moore et al., 2010; Song et al., 2017; Moner et al., 2018; Liu et al., 2020; Wang et al., 2020). A total number of 34 complete plastid genomes from Crassulaceae have been sequenced and deposited onto the GenBank database. *Kalanchoe* is one of the largest genera in this family, but little work has been done on the plastid genomes of *Kalanchoe*. There have been only two species with their plastomes sequenced and analyzed to date, and they are *Kalanchoe tomentosa* Baker (Zhao et al., 2020) and *Kalanchoe daigremontiana* Raym.-Hamet and H. Perrier (formerly known as *Bryophyllum daigremontianum* A. Berger) (Zhou et al., 2021). The lack of sequence data has impeded the progress in determining the structural variations among plastomes of different *Kalanchoe* species and interpreting the evolutionary history of this genus.

In this study, the complete plastid genomes of five *Kalanchoe* horticultural plants were sequenced and characterized, aiming the following: (1) to interpret the phylogenetic relationship of the five species based on plastome sequence analyses; (2) to explain why *Kalanchoe* is better adapted to dry environment at the molecular level based on evolutionary (positive selection) analyses. This study will deepen our understanding of what has happened to the genus *Kalanchoe* in the history of life and how it has gained the ability to cope with dry environments.

## Materials and Methods

### Preparation of Plant Materials, DNA Extraction, and Sequencing

Fresh leaves of *K. daigremontiana, Kalanchoe delagoensis* Eckl. and Zeyh. [Formerly known as *Bryophyllum delagoense* (Eckl. and Zeyh.) Druce], *Kalanchoe fedtschenkoi* Raym. -Hamet and H. Perrier [formerly as *Bryophyllum fedtschenkoi* (Raym.-Hamet and H. Perrier) Lauz.-March.], *Kalanchoe longiflora* Schltr. and *Kalanchoe pinnata* (Lam.) Pers. [formerly as *B. pinnatum* (Lam.) Oken] were collected from the greenhouse at Zhengzhou University (Henan, China) (Supplementary Material 1). Samples were flash-frozen and stored at −80°C until use. Total genomic DNA of the five samples was extracted with the Tiangen Plant Genomic DNA Kit (TIANGEN, Beijing, China) following the protocol provided by the manufacturer. The NanoDrop 2000 Spectrophotometer and Qubit 4 Fluorometer from Thermo Fisher Scientific, Wilmington, DE, USA were used to assess the quantity and quality of the total genomic DNA. DNA libraries were constructed using the Illumina Paired-End DNA Library Kit (Illumina Inc., San Diego, CA, USA) and sequenced employing the NovaSeq 6000 platform with a paired-end reading length of 150 bp (NovoGene Inc., Beijing, China).

### Genome Assembly and Annotation

The raw sequencing data were *de novo* assembled into complete plastid genomes with the GetOrganelle toolkit following a standard procedure (Jin et al., 2020; https://github.com/Kinggerm/GetOrganelle). Former published plastid genomic sequences of the family Crassulaceae were retrieved from the NCBI database and used as the seed database file. The five complete plastid genomes were annotated using Plastid Genome Annotator (Qu et al., 2019; https://github.com/quxiaojian/PGA) in reference to the plastid genome of *K. tomentosa* (Accession no. MN794319). Programs, including GeSeq (Tillich et al., 2017), HMMER (Wheeler and Eddy, 2013), tRNAscan-SE version 2.0.6 (Lowe and Eddy, 1997), and GB2sequin (Lehwark and Greiner, 2019) implemented in the CHLOROBOX online toolbox (https://chlorobox.mpimp-golm.mpg.de/geseq.html) were used to check the accuracy of annotation. Physical maps of the circular plastid genomes were generated and visualized using the online program Chloroplot (Zheng et al., 2020; https://irscope.shinyapps.io/chloroplot/).

### Comparison of the Five *Kalanchoe* Plastomes

Comparative analyses were performed between *K. tomentosa* plastome and the five newly sequenced plastomes. Genomic structures of these plastomes were analyzed and visualized using Irscope (Amiryousefi et al., 2018; https://irscope.shinyapps.io/irapp/).

To identify variable regions and intra-generic variations, the annotated plastomes were visualized using the Shuffle-LAGAN mode included in mVISTA (Frazer et al., 2004; https://genome.lbl.gov/vista/mvista/submit.shtml) with the *K. tomentosa* plastome as a reference. Features including large single copy (LSC), small single copy (SSC), inverted repeats (IRa and IRb), variable sites, and insertion or deletion (InDel) events across the five *Kalanchoe* plastomes were detected using DnaSP v6.10.03 (Rozas et al., 2017; http://www.ub.edu/dnasp/). Furthermore, nucleotide diversity (Pi) of plastomes among genus *Kalanchoe* and family Crassulaceae species (Supplementary Material 1) were calculated using the sliding window method with a window length of 600 bp and a step size of 100 bp.

Simple sequence repeats (SSRs) were identified using the MISA web (Beier et al., 2017; http://misaweb.ipk-gatersleben.de/) with the following criteria: 10, 5, 4, 3, 3, and 3 repeat units for mono-, di-, tri-, tetra-, penta-, and hexa-nucleotides, respectively. REPuter (Kurtz et al., 2001; https://bibiserv.cebitec.uni-bielefeld.de/reputer) was used to identify forward, palindrome, reverse, and complement repeated elements with a minimal length of 30 bp, an identity value of more than 90%, and a Hamming distance of 3.

### Phylogenetic Analyses

The five *Kalanchoe* plastomes were selected for phylogenetic analyses with some closely related genus (Supplementary Material 1). The species *Saxifraga stolonifera* Curtis (Accession no. MN496079) belonging to the family Saxifragaceae A. L. Jussieu served as the out-group. Complete plastome sequences and protein-coding genes (PCGs) were selected for phylogenetic analyses. A multiple sequence alignment was generated using MAFFT v7.467 (Katoh et al., 2002; https://mafft.cbrc.jp/alignment/software/). Phylogenetic analyses were conducted using the Maximum likelihood (ML) analysis implemented in the IQ-TREE software (Nguyen et al., 2014; http://www.iqtree.org/) and the Bayesian inference (BI) method in the MrBayes software (Huelsenbeck and Ronquist, 2001; http://nbisweden.github.io/MrBayes/). For the ML analysis, a bootstrap replicate of 50,000 was performed with the SH-aLRT branch test (Guindon et al., 2010). For the BI analysis, we performed two independent Markov Chain Monte Carlo chains with 2,000,000 generations. The first 25% of trees were discarded. The average SD of split frequencies is below 0.01. Both consensus trees were displayed using the online tool iTOL (Letunic and Bork, 2006; https://itol.embl.de/itol.cgi).

### Detection of Positive Selection on Plastidic Genes

Signs of positive selection were determined for each PCGs in the five *Kalanchoe* plastomes with EasyCodeML v1.31 (Gao et al., 2019; https://github.com/BioEasy/EasyCodeML), and the CodeML model implemented in the PAML package (Yang, 2007; http://web.mit.edu/6.891/www/lab/paml.html). The phylogenetic tree file was generated using the maximum likelihood approach as described in the PAML instruction manual. The ratio (ω) of the non-synonymous substitution rate (dN) to the synonymous substitutions rate (dS) for each PCG was calculated using the site model. The likelihood ratio test of M8a (beta and ω = 1) vs. M8 (beta and ω) was performed to find sites under positive selection. Both the Naive Empirical Bayes (NEB) and the Bayes Empirical Bayes (BEB) approaches (Yang et al., 2005) were employed to assess the posterior probability values.

## Results and Discussion

### Characterization of the Five *Kalanchoe* Plastomes

In this study, the five plastome sequence data were obtained using the NovaSeq 6000 platform. The amount of raw data retrieved ranges from 28,988,682 reads for *K. delagoensis* to 31,184,708 reads for *K. pinnata*. After trimming, an average of 85.48% of clean reads was obtained and used for *de novo* assembly (Table 1). The average depth of coverage was 433×, 479×, 417×, 465×, and 520× for the assembled plastid genome of *K. daigremontiana, K. delagoensis, K. fedtschenkoi, K. longiflora*, and *K. pinnata*, respectively. The final complete plastome sequence data were submitted to GenBank.

**Table 1:** Summary statistics for the assembly of the five *Kalanchoe* chloroplast genomes.

**Species**	***K. daigremontiana***	***K. longiflora***	***K. pinnata***	***K. fedtschenkoi***	***K. delagoensis***
Genome size (bp)	150,049	149,978	150,056	150,001	150,018
Large single copy (LSC; bp)	82,125	82,095	82,131	82,015	82,225
Inverted repeats (IR; bp)	25,469	25,413	25,469	25,487	25,392
Small single copy (SSC; bp)	16,986	17,057	16,987	17,012	17,009
Guanine-cytosine (GC) content (LSC/IR/SSC)	37.6% (35.6/42.9/31.4%)	37.6% (35.6/43.0/31.4%)	37.7% (35.7/42.9/31.5%)	37.7% (35.7/42.9/31.4%)	37.6% (35.6/43.0/31.4%)
Total number of genes (CDS/tRNA/rRNA)	131 (86/37/8)	131 (86/37/8)	131 (86/37/8)	131 (86/37/8)	131 (86/37/8)

**Figure 1:**
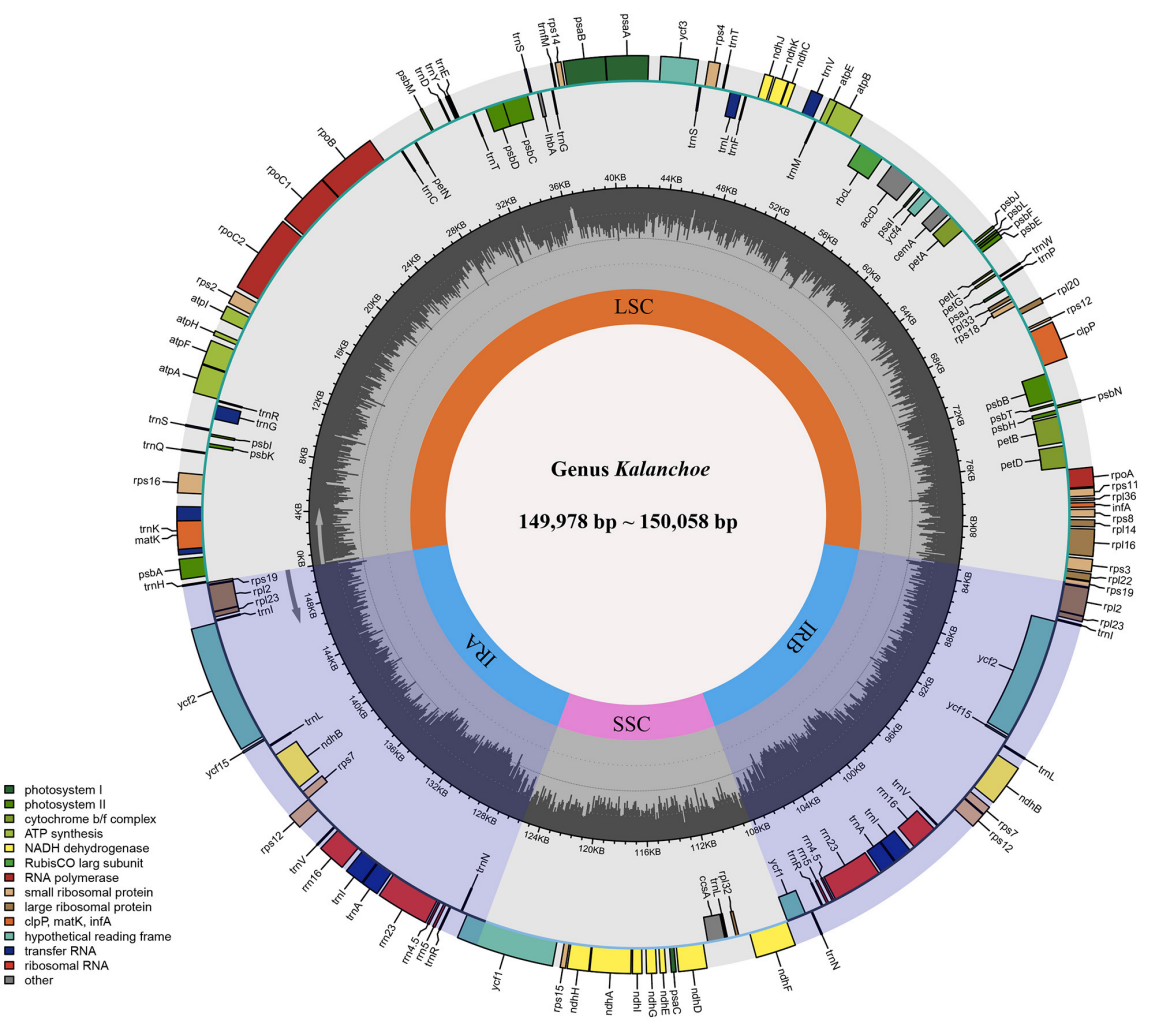
Gene map and genome organization of the five *Kalanchoe* plastomes. It exhibits a typical quadripartite structure with a pair of inverted repeat regions (IRA and IRB) separating the small single-copy region (SSC) and the large single-copy region (LSC). Functional genes are also included and color-coded in this map, and guanine-cytosine (GC) contents are drawn.

The five plastid genomes of *Kalanchoe* all possess a typical quadripartite structure, comprising one LSC region and one SSC region separated by a pair of IR regions (IRa and IRb) (Figure 1). This is consistent with the published plastome structures of species from the family Crassulaceae (Dong et al., 2013; Seo and Kim, 2018; Zhao and Zhang, 2018; Chang et al., 2020; Kim and Kim, 2020; Li and Chen, 2020). The size of these plastomes ranges from 149,978 bp in *K. longiflora* to 150,056 bp in *K. pinnata* (Table 1), which is similar to the size of plastomes from other genera of Crassulaceae (Zhao et al., 2020) with only a few exceptions. The five species have larger plastome sizes than *Sedum emarginatum* (149,118 bp) and *Sedum oryzifolium* (149,609 bp) (Chang et al., 2020; Li and Chen, 2020). The size of the LSCs ranges from 82,015 bp (*K. fedtschenkoi*) to 82,225 bp (*K. delagoensis*) with the estimated guanine-cytosine (GC) content to be 35.6%, whereas the size of the SSCs ranges from 16,986 bp (*K. daigremontiana*) to 17,057 bp (*K. longiflora*) with the GC content to be 31.4%, the size of the IRs ranges from 25,392 bp (*K. delagoensis*) to 25,487 bp (*K. fedtschenkoi*) with a GC content of 42.9%. The genome size and the GC contents are highly conserved among the five *Kalanchoe* species, which are also similar to the previously published species from the family Crassulaceae (Xu et al., 2015).

**Figure 2:**
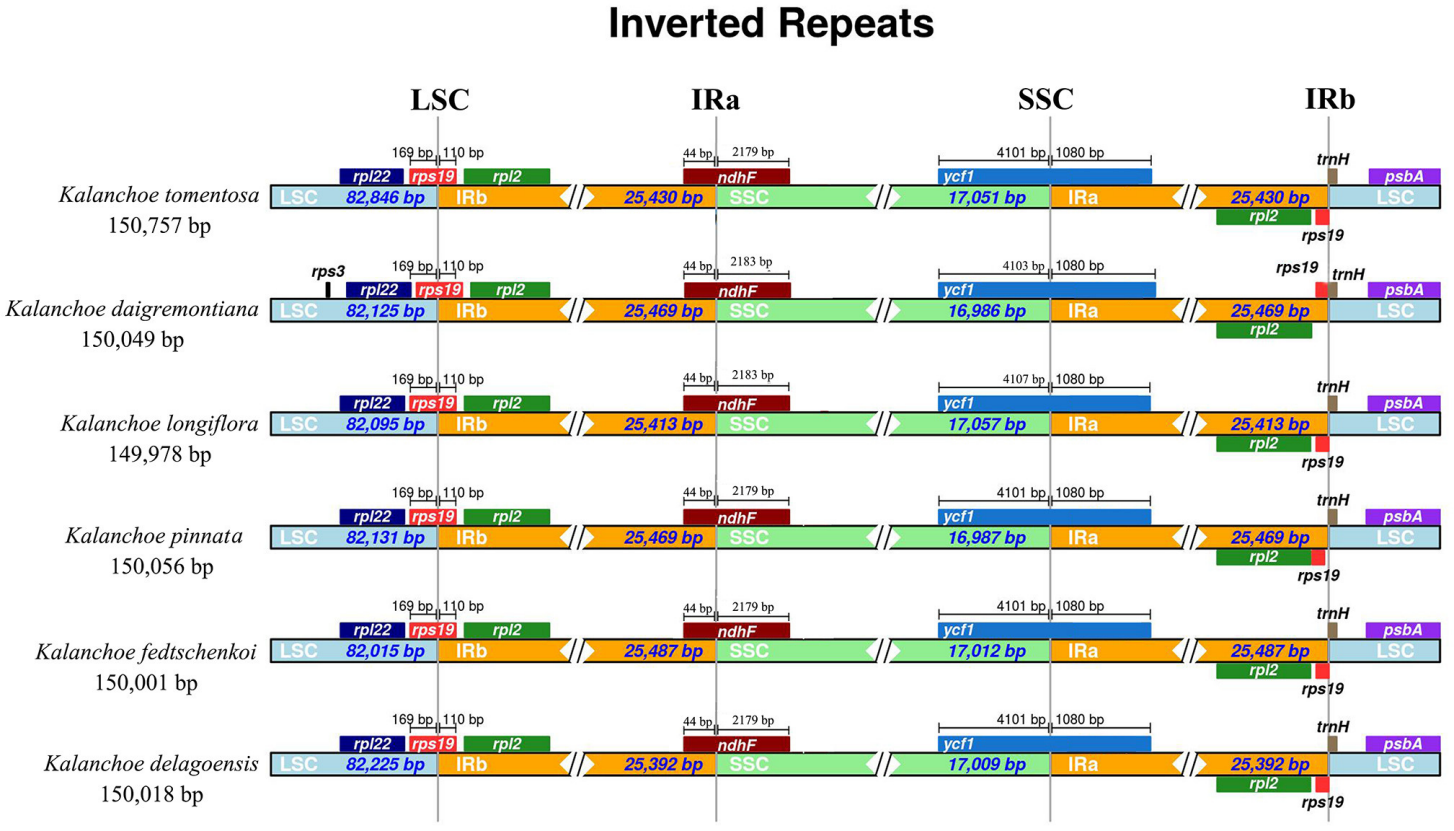
Comparison of the border positions of large single copy (LSC), inverted repeat B (IRB), small single copy (SSC), and inverted repeat A (IRA) regions across the five *Kalanchoe* plastomes. Functional genes and truncated fragments are denoted by colored boxes. The sizes of gene fragments located at boundaries are indicated by the base pair lengths.

All of the five plastid genomes of *Kalanchoe* encode 131 genes, containing 86 PCGs, 37 tRNAs, and eight rRNAs (Figure 1). The gene number of each species is similar to that of *Rhodiola* and other species including *Hylotelephium, Orostachys, Phedimus, Rosularia*, and *Sinocrassula* (Zhao et al., 2020). These plastomes contain more genes than that of *Hylotelephium verticillatum* (128 genes) (Kim and Kim, 2020) and *Sedum sarmentosum* (113 genes) (Dong et al., 2013). Among all the functional genes, eight PCGs, seven tRNAs, and four rRNAs were found to be duplicated in the IR regions. The distinct junction sites were found between the IR boundaries within the LSC (IRb: *rps19*; IRa: *rps19*-*trnH*) and SSC (IRb: *ndhF*; IRa: *ycf1*) regions. Each site is present at a particular position and in a particular length in reference to *K. tomentosa* (Figure 2). The potential pseudogene *rps12* has the duplicated 3′ end in the IR and the 5′ end in the LSC. The identification of distinct junction sites in these plastomes shows that the LSC/IRa boundary is located in the *rps12* gene, and a similar pattern was reported in *Rhodiola* (Zhao et al., 2020). In addition, the SSC/IRb boundary in *Rhodiola* is located in the intergenic spacer region between *ndhF* and *ycf1*, and this is different from *Kalanchoe*, which has its SSC/IRb boundary located in the *ndhF* gene (Figure 2). A few comparative genomic studies have proven that IR/LSC or IR/SSC boundary shifts (Wang et al., 2017; Gao et al., 2018; Bedoya et al., 2019; Zavala-Páez et al., 2020) are common in angiosperms and that the instability of distinct junctions was very important in shaping genome structures during plastome evolution (Maréchal and Brisson, 2010; Xu et al., 2015; Zhu et al., 2016).

### Comparison of the Five *Kalanchoe* Plastomes

**Figure 3:**
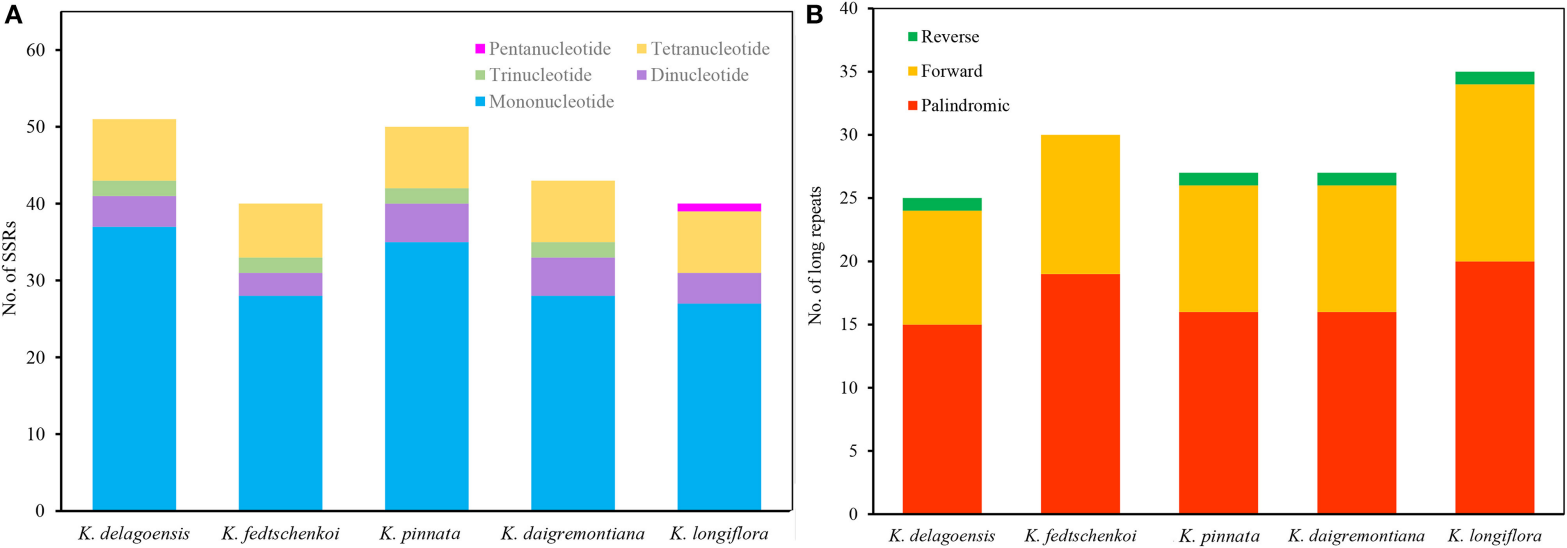
Analysis of simple sequence repeats (SSRs) **(A)** and long repeats **(B)** of the five *Kalanchoe* plastomes. **(A)** The number of SSRs with different types, including mono-, di-, tri-, tetra-, and pentanucleotides were analyzed among the five *Kalanchoe* species. **(B)** The number of long repeats with different types (reverse, forward, and palindromic) present in the five plastomes were also analyzed.

In this study, we detected 224 SSRs in the five *Kalanchoe* plastomes. Most SSRs were located in the LSC regions. The number of SSRs per species ranges from 40 (*K. longiflora* and *K. fedtschenkoi*) to 51 (*K. delagoensis*). There are 25 to 37 mononucleotide repeats, three to five dinucleotide repeats, two trinucleotide repeats, and seven to eight tetranucleotide repeats in each plastome (Figure 3A). Trinucleotide repeats are absent from the plastome of *K. longiflora*, whereas pentanucleotide repeats are absent in other species. A total of 144 long repeats (>30 bp) were identified in the five plastid genomes, including 86 palindromic repeats, 54 forward repeats, and four reverse repeats (Figure 3B). Most repeats are shorter than 100 bp, whereas the four palindromic repeats found in *K. longiflora* are 1,180, 5,671, 8,885, and 9,674 bp in length, indicating a complex structure in the IR regions.

The multiple sequence alignment of the five *Kalanchoe* plastomes with a length of 151,763 bp revealed a total number of 1,056 polymorphic sites (Pi = 0.00313), including 984 singleton variable sites and 72 parsimony informative sites (Table 2; Supplementary Material 2). Among these polymorphic sites, 490 are positioned in the intergenic regions, 130 are in the introns, and 436 are in the exons (Table 2). Singleton variable sites are abundant in the five plastomes, and a total number of 361 transversions (Tv) and 57 transitions (Ts) were counted in the gene coding regions (Table 3). Among the 57 Ts, 35 are between G and C, whereas 22 are between A and T (Supplementary Material 2). In addition, the 361 Tv are related to changes in the GC contents in different species. For the 436 polymorphic sites in the gene coding regions, only synonymous substitutions (S) were detected in genes related to photosynthesis. Its rate (dS) is comparably low and equals to the rate of having non-synonymous substitutions (dN) in self-replicating genes or other genes (Supplementary Material 2). Similar results are also reported in *Machilus* (Song et al., 2015). However, a different pattern was observed in *ycf1* and *ycf2*. The dS of *ycf1* and *ycf2* is relatively higher than dN, supporting the conservation of the photosynthesis-related genes in the plastomes. There are 354 InDel mutations in these five plastomes with 289 in the intergenic regions, 49 in introns, and 16 InDels present in the exons of *accD, matK, ycf1*, etc (Supplementary Material 2). Furthermore, among these InDel mutations, 118 are SSR InDels, and 34 InDels contain singleton variable sites (Table 2). In addition, InDel mutations occurring in the coding regions are more likely to cause changes to structures of the PCGs. For example, four independent InDel events to *ycf2* in *K. fedtschenkoi* have led to an extra length of seven amino acids in the final amino acid product of this gene (Supplementary Material 2).

**Table 2:** Summary statistics for variable sites and insertion or deletion (InDel) mutations of the five *Kalanchoe* plastomes.

	**Polymorphic sites**	**InDels events**
Total	1,056	354
SSRs	–	118
Singleton variable sites	984	34
Intergenic	490	289
Intron	130	49
Extron	436	16
Parsimony informative sites	72	–

**Table 3:** Comparisons of mutations, including the number of transversions (Tv), transitions (Ts), synonymous (S), and non-synonymous (N) substitutions per protein-coding genes across the plastomes of the five *Kalanchoe* species.

**Category**	**Group of genes**	**Gene**	**Tv**	**Ts**	**S**	**N**
Photosynthesis	ATP synthase	atpA	11	0	10	1
		atpE	9	1	8	2
		atpF	6	0	1	5
		atpI	8	2	9	1
	Cytochrome b6/f complex	petA	5	0	3	2
		petB	3	2	5	0
		petD	1	0	1	0
	NADH oxidoreductase	ndhA	7	0	4	3
		ndhC	1	0	0	1
		ndhD	7	1	5	3
		ndhE	1	1	2	0
		ndhF	15	4	8	11
		ndhG	6	0	4	2
		ndhH	10	1	9	2
		ndhI	2	1	3	0
		ndhJ	2	0	1	1
		ndhK	4	0	0	4
	Photosystem I	psaA	3	1	4	0
		psaB	7	1	7	1
		psaJ	1	0	1	0
		ycf3	4	0	4	0
		ycf4	3	0	3	0
	Photosystem II	psbA	5	0	4	1
		psbB	4	2	6	0
		psbC	7	1	7	1
		psbD	2	0	1	1
		psbE	1	0	1	0
		psbF	1	1	2	0
		psbI	1	0	1	0
		psbJ	1	0	1	0
		psbK	2	0	0	2
		psbT	1	0	1	0
	Rubisco	rbcL	5	0	5	0
		**Total**	**146**	**19**	**121**	**44**
Self-replication	DNA dependent RNA polymerase	rpoA	8	1	2	7
		rpoB	12	0	8	4
		rpoC1	13	2	9	6
		rpoC2	26	5	8	23
	Large subunit of ribosomal proteins	rpl16	1	0	0	1
		rpl2	1	0	1	0
		rpl20	2	0	1	1
		rpl22	8	1	2	7
		rpl36	1	0	0	1
	Small subunit of ribosomal proteins	rps11	1	0	1	0
		rps15	3	1	2	2
		rps16	1	0	0	1
		rps19	3	0	0	3
		rps2	1	0	1	0
		rps3	3	3	4	2
		rps4	2	0	1	1
		rps8	3	2	1	4
		**Total**	**89**	**15**	**41**	**63**
Other gene	c-type cytochrom synthesis gene	ccsA	8	2	4	6
	Envelop membrane protein	cemA	7	0	2	5
	Maturase	matK	24	9	9	24
	Subunit acetyl-CoA-arboxylase	accD	10	1	1	10
		**Total**	**49**	**12**	**16**	**45**
Unknown gene	Conserved open Reading frames	ycf1	67	9	55	21
		ycf2	10	2	1	11
		**Total**	**77**	**11**	**56**	**32**

**Figure 4:**
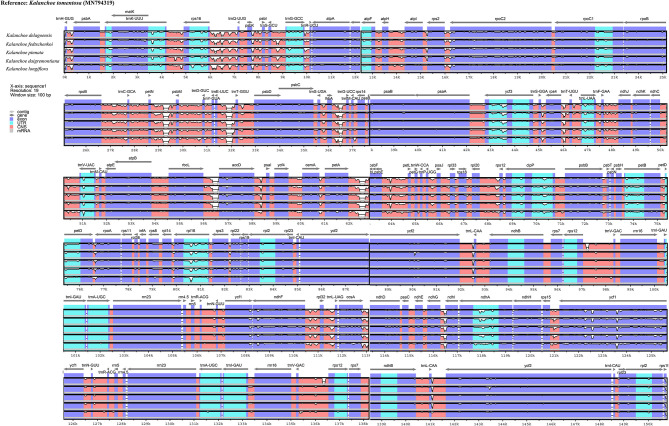
Sequence identity plot comparing the five plastomes using mVista with *Kalanchoe tomentosa* (Accession no. MN794319) as a reference. The *y*-axis corresponds to percentage identity (50–100%), while the *x*-axis shows the position of each region within the locus. Arrows indicate the transcription of annotated genes in the reference genome.

**Figure 5:**
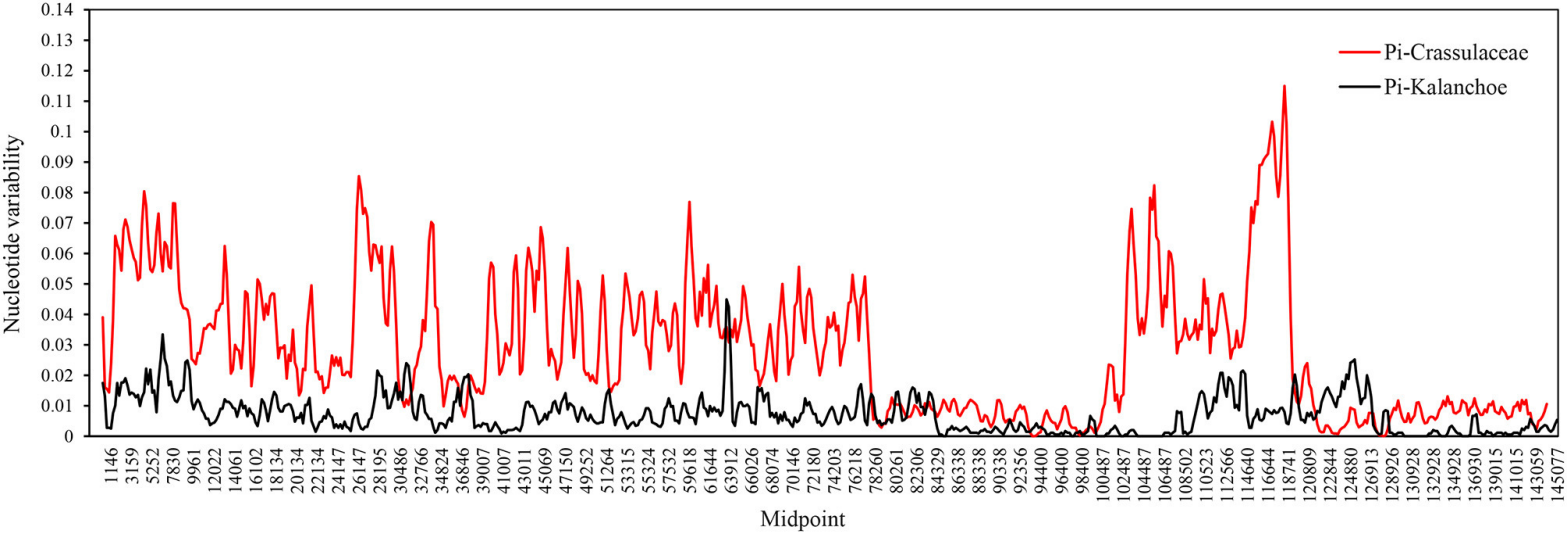
Sliding window analysis between the *Kalanchoe* plastome (black line) and the family Crassulaceae plastome (red line). Window size: 600 bp, step size: 200 bp. *X*-axis: the position of the midpoint of a window (kb). *Y*-axis: nucleotide diversity of each window.

Interspecific comparison among the five *Kalanchoe* species was established and plotted using mVISTA with the annotated *K. tomentosa* plastome as a reference (Figure 4). A single universal DNA marker is not sufficient in a large-scale phylogenetic study, especially in studies concerning closely related taxa (Li et al., 2015). Comparing the five plastomes, highly divergent mutations occur in the *trnH*-*psbA, matK, rps16*-*trnQ, trnE*-*trnT, rpoC1*-*rpoB, ycf1, ccsA*-*ndhD*, and *ndhG*-*ndhI* regions. This is similar to that in *Rhodiola*, containing highly divergent sequences in non-coding regions (Zhao et al., 2020). The comparison among these plastomes shows that sequences are more conserved in the IR regions (Pi = 0.00172) than that of the LSC (Pi = 0.00935) and SSC (Pi = 0.01249) regions. Results of the sliding window analysis also suggest that a highly variable mutation is present in the IR regions across all five species from the genus *Kalanchoe* or from the family Crassulaceae in general (Figure 5). As expected, our study suggests that sequences in the IR regions are less variable than that in the LSC or SSC regions and that sequences in the coding regions are also more conserved than that in the intergenic regions (Walker et al., 2014; Abdullah et al., 2019; Xue et al., 2019). Phylogenetic studies of species from the family Crassulaceae were generally done using *matK, rpl16, trnL-F, psbA, psbA*-*trnH, rps16*, and nuclear ITS as molecular markers (Mort et al., 2001, 2005; Acevedo-Rosas et al., 2004; Fairfield et al., 2004; Gontcharova et al., 2006; Carrillo-Reyes et al., 2008; Zhang et al., 2014; Nikulin et al., 2016; Ito et al., 2017), and some major crown clades of Crassulaceae have been defined. However, the evolutionary relationship of this family has not been fully elucidated. The highly variable regions identified in this study, containing either SNPs or InDels, would serve prominent roles in identifying potential DNA barcoding markers in further systematic and phylogenetic studies of the family Crassulaceae.

### Evolutionary Analyses of the Five *Kalanchoe* Species

**Figure 6:**
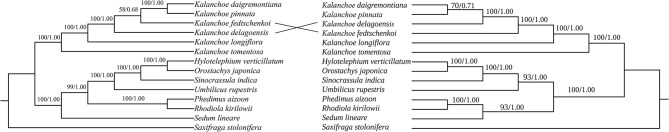
Phylogenetic reconstruction of the *Kalanchoe* genus using the Complete chloroplast genome sequences (left; **A**) and PCQ sequences (right; **B**) data with *Saxifraga stolonifera* as the outgroup for rooting. Bootstrap value/posterior probability are shown on the corresponding branch.

The phylogenetic trees generated with either the five whole plastomes or the PCGs are identical in topology for both ML and BI methods (Figure 6). *Kalanchoe* and *Bryophyllum* are grouped in one clade in both trees with high bootstrap values, and this is consistent with the result of the phylogenetic study based on *matK* (Mort et al., 2001). This grouping is further supported by a morphological similarity shared by *Bryophyllum* and *Kalanchoe*, they both possess fused corolla (Gehrig et al., 2001; Mort et al., 2001).

The inferred phylogenetic relationship of the five *Kalanchoe* species is highly correlated with their morphology with only a few exceptions (BS = 100; PP = 1.00). *K. tomentosa* and *K. longiflora* are both placed in the *Kalanchoe* clade (Gehrig et al., 2001) even though they demonstrate different leaf and stem morphologies. *K. daigremontiana, K. pinnata, K. fedtschenkoi*, and *K. delagoensis*, which were formerly classified as *Bryophyllum* (Smith and Figueiredo, 2018), are now present in the *Kalanchoe* clade (Figures 6A,B). Being different from the ITS analysis (Gehrig et al., 2001), a closer phylogenetic relationship can be referred to between *K. daigremontiana* and *K. fedtschenkoi*, and both of them have sessile leaves and toothed apex (Baldwin, 1938). The phylogenetic relationship among *K. daigremontiana, K. pinnata*, and *K. fedtschenkoi* was weakly supported in this study. In addition, we inferred that morphological similarities among different species could be due to interspecific hybridization and rapid radiation of this genus (Baldwin, 1938), which might have led to species delimitation under certain circumstances (Izumikawa et al., 2008; Zhao et al., 2020).

Likelihood ratio test employing the M8a vs. M8 model would produce slightly biased results in adaptive evolution analysis (Wong et al., 2004; Berlin and Smith, 2005). In this study, sites under positive selection were identified in the five *Kalanchoe* plastomes (Supplementary Material 3). Eleven genes with sites under positive selection were identified using both the NEB and BEB methods (Table 4). Those genes are *ccsA, clpP, ndhD, ndhF, ndhI, psbL, rbcL, rpoA, rps8, rps15*, and *ycf1*. The number of positively selected sites ranges from 1 to 6 in each gene. The 11 genes are thought to be essential to processes related to photorespiration (Shikanai et al., 2001; Kikuchi et al., 2013) and may have been involved in the evolution of the CAM pathway. *Kalanchoe* species with the CAM photosynthetic pathway (Gehrig et al., 2001) are generally distributed in dry habitats, indicating that *Kalanchoe* species have adopted a better way of fixing CO2. It has also made them more adaptive to extreme dry environments in Madagascar and Africa. The role of these genes in the adaptation of *Kalanchoe* to a dry environment is further supported by the fact that these genes are not under positive selection pressure in the genus *Rhodiola* distributed in an alpine environment (Zhao et al., 2020).

**Table 4:** Detection of sites under positive selection using the likelihood ratio test.

**Genes**	**Model**	**Ln L**	**Estimates of parameters**	**Models compared**	**2ΔLNL**	**LRT *p*-value**	**Positively selected sites**
ccsA	M8	−2,260.95	p0 = 0.94 p = 25.44 q = 99.00 (p1 = 0.06) w = 2.77				
	M8a	−2,264.82	p0 = 0.82 p = 14.87 q = 99.00 (p1 = 0.18) w = 00	M8a vs. M8	7.75	0.01	168 H^**^ 174 P^*^
clpP	M8	−1,043.18	p0 = 0.99 p = 0.33 q = 1.55 (p1 = 0.01) w=55.05				
	M8a	−1,049.96	p0 = 0.88 p = 4.00 q = 65.89 (p1 = 0.12) w = 1.00	M8a vs. M8	13.55	0.00	43 S^**^
ndhD	M8	−3,316.08	p0 = 1.00 p = 0.04 q = 0.20 (p1 = 0.00) w = 8.97				
	M8a	−3,319.73	p0 = 0.93 p = 0.51 q = 7.82 (p1 = 0.07) w = 1.00	M8a vs. M8	7.31	0.01	1 M^**^
ndhF	M8	−3,460.77	p0 = 0.99 p= 0.29 q= 1.24 (p1= 0.01) w= 4.44				
	M8a	−3,464.42	p0 = 0.87 p = 2.44 q = 24.04 (p1 = 0.12) w = 1.00	M8a vs. M8	7.31	0.01	215 F^*^ 484 Q^**^
ndhI	M8	−1,009.15	p0 = 0.98 p = 0.04 q = 0.28 (p1 = 0.02) w = 5.29				
	M8a	−1,009.75	p0 = 0.90 p = 0.01 q = 0.28 (p1 = 0.09) w = 1.00	M8a vs. M8	1.21	0.27	161 V^**^
psbL	M8	−187.74	p0 = 0.94 p = 0.01 q = 2.38 (p1 = 0.06) w = 15.22				
	M8a	−192.14	p0 = 0.84 p = 0.01 q = 99.00 (p1 = 0.16) w = 1.00	M8a vs. M8	8.81	0.00	1 T^**^ 10 N^*^
rbcL	M8	−2,579.79	p0 = 0.97 p = 0.15 q = 3.55 (p1 = 0.03) w = 2.70				
	M8a	−2,581.89	p0 = 0.92 p = 0.01 q = 3.23 (p1 = 0.08) w = 1.00	M8a versus M8	4.20	0.04	28 E^*^ 97 Y^*^ 225 I^**^ 228 S^**^ 328 S^*^ 429 Q^*^ 475 I^*^
rpoA	M8	−2,316.12	p0 = 0.99 p = 0.15 q = 0.46 (p1 = 0.01) w = 17.07				
	M8a	−2,328.18	p0 = 0.82 p = 7.55 q = 99.00 (p1 = 0.18) w = 1.00	M8a versus M8	24.12	0.00	311 R^**^ 333 L^**^
rps8	M8	−884.00	p0 = 0.98 p = 1.80 q = 4.03 (p1 = 0.02) w = 9.24				
	M8a	−888.62	p0 = 0.78 p = 15.02 q = 99.00 (p1 = 0.22) w = 1.00	M8a vs. M8	9.24	0.00	52 R^**^ 56 N^**^
rps15	M8	−695.64	p0 = 0.95 p = 0.74 q = 3.83 (p1 = 0.05) w = 5.44				
	M8a	−701.07	p0 = 0.86 p = 7.26 q = 99.00 (p1 = 0.14) w = 1.00	M8a vs. M8	10.85	0.00	7 F^**^ 33 N^*^ 67 V^**^
ycf1	M8	−12,401.09	p0 = 0.98 p = 0.20 q = 0.22 (p1 = 0.02) w = 4.86				
	M8a	−12,423.25	p0 = 0.60 p = 12.56 q = 99.00 (p1 = 0.40) w = 1.00	M8a vs. M8	44.33	0.00	445 N^**^ 542 R^**^ 592 M^**^ 672 V^**^ 953 D^**^ 1080 Q^**^ 1295 S^*^

*Posterior probability values were assessed with both Naive Empirical Bayes (NEB) and Bayes Empirical Bayes (BEB) approaches*.

* *Denotes positively selected sites supported by either NEB or BEB method (p < 0.05)*.

** *Denotes positively selected sites supported by both NEB and BEB methods (p < 0.05)*.

## Conclusion

In conclusion, the complete plastid genomes of the five *Kalanchoe* species, such as *K. daigremontiana, K. delagoensis, K. fedtschenkoi, K. longiflora*, and *K. pinnata*, were sequenced and analyzed. In general, the size, structure, GC contents, and the number of genes are similar to other Crassulaceaen plastid genomes. Despite these similarities, several SSRs and divergent regions were detected, and they were used in interpreting the evolutionary relationship of the five species. Phylogenetic analyses based on these five whole plastomes and the PCGs produced well-supported values, which allow us to infer the phylogenetic relationship of these species with high confidence levels. Our results support that *Bryophyllum* and *Kalanchoe* should be treated as one single genus and that they have evolved a series of positively selected genes related to the CAM photosynthetic carbon assimilation pathway. These findings suggest that the plastid genome can be used as an informative genetic marker for improving our understandings of speciation and diversification in *Kalanchoe*, most of which are drought-resistant succulent plants.

## Data Availability Statement

The datasets presented in this study can be found in online repositories. The names of the repository/repositories and accession number(s) can be found in the article/Supplementary Material.

## Author Contributions

XT and YS conceived the idea and designed the research. XZ and TS conducted the experiments. XZ, YM, and KM conducted the collection of specimens. XT and JG wrote the manuscript. All authors contributed to the article and approved the submitted version.

## Conflict of Interest

The authors declare that the research was conducted in the absence of any commercial or financial relationships that could be construed as a potential conflict of interest.

## Publisher's Note

All claims expressed in this article are solely those of the authors and do not necessarily represent those of their affiliated organizations, or those of the publisher, the editors and the reviewers. Any product that may be evaluated in this article, or claim that may be made by its manufacturer, is not guaranteed or endorsed by the publisher.
